# Implementing healthy food policies in health sector settings: New Zealand stakeholder perspectives

**DOI:** 10.1186/s40795-024-00924-z

**Published:** 2024-09-07

**Authors:** Magda Rosin, Cliona Ni Mhurchu, Sally Mackay

**Affiliations:** 1 Department of Epidemiology and Biostatistics, School of Population Health, Faculty of Medical and Health Sciences, University of Auckland, Private Bag 92019, Auckland Mail Centre, Auckland, 1142 New Zealand; 2 Centre for Translational Health Research: Informing Policy and Practice (TRANSFORM), Faculty of Medical and Health Sciences, University of Auckland, Auckland, New Zealand

**Keywords:** Food policy, Nutrition policy, Healthy eating, Hospital, Food environment, Food providers, Implementation, Employee, Workplace, Interviews

## Abstract

**Background:**

In 2016, a voluntary National Healthy Food and Drink Policy was released to improve the healthiness of food and drinks for sale in New Zealand health sector organisations. The Policy aims to role model healthy eating and demonstrate commitment to health and well-being of hospital staff and visitors and the general public. This study aimed to understand the experiences of hospital food providers and public health dietitians/staff in implementing the Policy, and identify tools and resources needed to assist with the implementation.

**Methods:**

A maximum variation purposive sampling strategy (based on a health district’s population size and food outlet type) was used to recruit participants by email. Video conference or email semi-structured interviews included 15 open-ended questions that focused on awareness, understanding of, and attitudes towards the Policy; level of support received; perceived customer response; tools and resources needed to support implementation; and unintended or unforeseen consequences. Data was analysed using a reflexive thematic analysis approach.

**Results:**

Twelve participants (eight food providers and four public health dietitians/staff) were interviewed; three from small (< 100,000 people), four from medium (100,000-300,000 people) and five from large (> 300,000 people) health districts. There was agreement that hospitals should role model healthy eating for the wider community. Three themes were identified relating to the implementation of the Policy: (1) Complexities of operating food outlets under a healthy food and drink policy in public health sector settings; (2) Adoption, implementation, and monitoring of the Policy as a series of incoherent ad-hoc actions; and (3) Policy is (currently) not achieving the desired impact. Concerns about increased food waste, loss of profits and an uneven playing field between food providers were related to the voluntary nature of the unsupported Policy. Three tools could enable implementation: a digital monitoring tool, a web-based database of compliant products, and customer communication materials.

**Conclusions:**

Adopting a single, mandatory Policy, provision of funding for implementation actions and supportive tools, and good communication with customers could facilitate implementation. Despite the relatively small sample size and views from only two stakeholder groups, strategies identified are relevant to policy makers, healthcare providers and public health professionals.

**Supplementary Information:**

The online version contains supplementary material available at 10.1186/s40795-024-00924-z.

## Background

Healthy food and drink policies and initiatives in public sector settings have an extensive population reach [1], can reverse some structural drivers of unhealthy diets [2], and promote healthier population diets [3]. Environmental interventions provide opportunities and remove barriers to healthy eating in the local food environments and can complement other initiatives to improve population health, such as changes in the overall food supply [1, 2]. There is growing evidence for the effectiveness of adopting and implementing a spectrum of comprehensive and evidence-informed food environment policies to positively influence population health outcomes [3].

Public healthcare facilities have a role in modelling health-promoting behaviours, and staff and visitors generally support interventions to positively influence hospital food environments [4]. The general public has been critical of the availability of unhealthy food and drink options in healthcare settings [5]. The presence of fast food outlets on some hospital premises led visitors to not only buy more fast foods but also perceive these foods as healthier compared to visitors in hospitals without fast food outlets [6]. The association of unhealthy food with health through hospitals is likely to also influence public perception and purchasing behaviours in the broader community [7], although by adopting and implementing healthy food policies, healthcare settings could have positive impact on healthier food purchasing overall.

Workplace dietary interventions can reach a large number of adults who typically may not engage in other health-promoting or disease-prevention programmes [8]. Overall, dietary interventions at work have been shown to increase consumption of easily accessible fruits and vegetables [9] and moderately improve fruit, vegetable and total fat consumption [10]. Improving hospital food environments is then an investment in the health and wellbeing of healthcare workers [11] who will likely eat at least some meals and snacks at work [12]. Easy access to unhealthy items creates challenges to healthier eating among healthcare staff in a stressful work environment, although ready availability and proximity of healthy choices can promote healthier diets [13, 14].

Several countries, states, and territories have introduced healthy food policies covering outlets and vending machines in healthcare facilities [15]. It is unknown how many policies listed in the NOURISHING Database [15] have been fully implemented since comprehensive auditing of food environments may be costly and resource-intensive [3, 16]. However, evaluations of the healthiness of food and drinks for sale in public sector healthcare facilities have been increasing [17], with findings indicating an increase in the proportion of healthy options. For example, mandatory and supported policies led to a significant reduction in the availability of sugar-sweetened beverages in New South Wales, Australia [18], and a substantial reduction in chocolate products on display in Scotland [19].

In New Zealand (NZ), District Health Boards (DHBs) were, until recently, responsible for providing healthcare services, primarily through hospitals and clinical centres. In 2022, as part of a national health system reform, the 20 DHBs were disestablished, and the provision of health services was centralised under the newly established Te Whatu Ora – Health New Zealand agency [20] that directly employs around 90,000 people (mainly nurses, doctors, allied and scientific staff, and corporate employees) [21], making it a major employer in NZ.

In 2015, the National DHB Food and Drink Environments Network (the Network), consisting of public health nutrition professionals, was formed to develop a consistent healthy food and drink policy for use in the 20 DHBs, because up until then DHBs had been developing and implementing different food and drink criteria independently [22]. The Network consisted of public health professionals and dietitians representing their respective DHBs, and nutrition and public health advisors from the Ministry of Health [23]. Additionally, the Heart Foundation, Activity & Nutrition Aotearoa (NZ), the Ministry for Primary Industries, the New Zealand Beverage Guidance Panel (NZ university researchers group advocating for a reduction in sugar-sweetened beverage consumption), and a University of Auckland academic (author Ni Mhurchu) provided support and advice during the Policy development [23]. The National Healthy Food and Drink Policy (the Policy) was finalised in December 2015, published by the Ministry of Health in September 2016, and was expected to be implemented over the next two years [23]. A limited review of key issues was carried out in 2019 to make the Policy more feasible, easy, and practical to implement [24]. A second edition of the Policy was published in September 2019 [24], but no future reviews were outlined at that stage.

The Policy was introduced to support organisations to provide healthy options to their staff and visitors (in-patient meals and food brought on-site for own consumption were excluded) in accordance with the national dietary guidelines [25]. The Policy aims to demonstrate commitment to health and well-being of staff, visitors and the general public, role model healthy eating, and provide one set of criteria for food industry operating in the health sector [24]. A customised traffic light system is outlined in the Policy to differentiate *Green* (*healthy*, high nutritional quality items), *Amber* (*less healthy* items providing some nutritional value), and *Red* (*unhealthy* items with a poor nutritional profile) foods and drinks. According to the Policy, Green items should make up at least 55% of all available choices, and Red items, such as confectionery, sugar-sweetened beverages (SSBs), and deep-fried items, are not permitted [24]. No tools or resources were developed to support food providers in implementing the Policy [26]. The Policy was not officially endorsed by the Ministry of Health, and the DHBs were encouraged, but not mandated, to adopt the Policy. In 2017, only five DHBs had adopted the Policy [27], with the number rising to eight in 2021 [28], while the remaining DHBs opted to continue with their own policies or were working towards adoption of the Policy.

Comprehensive monitoring of local and national policy adoption and implementation is important to identify and understand factors that can be adapted and refined to influence food policy actions and advocate for positive health outcomes [29]. The HealthY Policy Evaluation (HYPE study) was the first comprehensive evaluation of the adoption [28], implementation and impact of the voluntary Policy in NZ. As part of the HYPE evaluation, this study aimed to understand the experiences of implementing the voluntary Policy in New Zealand. The objectives of this study were to conduct key stakeholder interviews with hospital food providers and public health dietitians/staff (Network members) to understand barriers and facilitators to implementation of the Policy in their organisations and to identify tools and resources that could assist with the implementation.

## Methods

Ethical approval was granted by the Auckland Health Research Ethics Committee (reference number AH2519). Subsequent locality approvals were sought from individual DHBs to undertake evaluations on their respective sites, with one DHB declining to participate. The study is reported in line with the Consolidated Criteria for Reporting Qualitative Research (COREQ) guidelines [30].

### Study design

This study used a qualitative case study approach [31], underpinned by pragmatism as the research paradigm [32]. Pragmatic qualitative case studies are a valuable research tool to identify “solutions to real-world problems” [32 (p.34)] and to infer “general lessons learned from studying the case” [32 (p.98)]. Additionally, pragmatism often involves engagement between researchers and project stakeholders, including practitioners and policy makers, allowing research outcomes to be practical, useful and directly applicable to policy and practice [33].

### Participant selection and recruitment

Potential participants, i.e., food providers and members of the Network within any of the DHBs (collectively referred to as ‘the implementers’ from here on), were identified through the Network. These key informants were selected because they were likely to provide rich data on their experiences with implementing the Policy, from the perspective of food outlet operators and from an organisational viewpoint. Eligible food providers were retailers and operators of staff cafeterias, coffee shops, franchised outlets, or vending machines. The authors did not have previous connections with any food providers. However, CNM and MR were members of the Network, and all authors know some Network members through other professional contexts.

A maximum variation purposive sampling strategy was used to recruit participants [31]. The sampling variables were DHB population size (small < 100,000 people; medium 100,000-300,000 people; and large > 300,000 people [34]), and, additionally for food providers, the type of food outlet. One author (MR) approached all nominated potential participants up to three times by email between December 2021 and July 2022, providing detailed information about the study. Those who agreed to participate were sent a consent form, which they signed and returned by email. All participants had the option to have their whānau (family) present during the interview. Participants were reimbursed with a NZ$50 grocery voucher. All participants were informed about confidentiality and anonymity on the consent form. Personal information, including the name of the DHB and food outlet, was removed and anonymised in all research data, documentation, and reporting for this study, except on the signed consent form. Data were stored on secure, password-protected servers with access limited to the HYPE research team.

### Data collection

A semi-structured interview guide, adapted from the Queensland Health A Better Choice 2009 evaluation tools with permission (©State of Queensland), was chosen because it was previously utilised in a study using thematic analysis, closely aligned with our study’s methodological approach, and featured open-ended questions with prompts, facilitating in-depth exploration of participants’ perspectives. To align the interview guide with the aims of this study, findings from our previous work on the implementation of healthy food and drink policies in public sector workplaces [26, 35] were used to refine the interview questions and associated prompts.

The language was adapted to suit the NZ context and participants in this study, with prompts for each question based on those included in Queensland Health’s guide and the findings from two previous reviews conducted by our team [26, 35]. Questions about participants’ backgrounds, awareness and attitudes towards the Policy, and further work and resources were largely retained. However, questions regarding how well the Policy was implemented were omitted, as the availability of Green, Amber and Red options had been objectively assessed in another part of the HYPE study. Our guide also included questions about practical actions taken to implement the Policy, responses from staff and visitors, and any unintended or unforeseen consequences of adopting and implementing the Policy. The interview guide (Additional File 1) consisted of 15 questions and was designed to assess the experiences of key informants in implementing the Policy; awareness, understanding, and attitudes towards the Policy; level of support received; perceived customer response to the Policy; tools and resources needed to support implementation; and any unintended or unforeseen consequences.

In-depth video conference interviews (Zoom software, zoom.us or Microsoft Teams platform) were scheduled between February and July 2022 and conducted by one author (MR), who is trained in qualitative research methods. Face-to-face interviews were not conducted due to the Covid-19 restrictions at the time. At the beginning of the interview, the research purpose, process, and participants’ rights were explained, any questions answered, and permission to audio record the interviews (as previously agreed to on the consent form) was verbally confirmed.

All interviews were transcribed verbatim by MR and participants were given the opportunity to review and edit their transcripts. An option to complete the interview questions by email was also offered from May until July 2022, recognising that Covid-19 significantly impacted food providers and staff operating in healthcare facilities, some of whom needed more flexibility to participate in the study. Email participants received the same interview guide and compensation, and clarification and further details were provided where necessary. Email interviewing can produce rich, in-depth data since participants have time to consider each question as interviews are self-paced [36, 37].

### Data analysis

One author (MR) led the data analysis using a research question-led reflexive thematic analysis approach [38]. The inductive and iterative six-step process was guided by pragmatism and no framework or model was used during analysis. First, MR became familiar with the data by conducting the interviews, transcribing, and reading the entire dataset. Second, interview transcripts were analysed using QSR’s NVivo software for qualitative data analysis. One coded transcript was shared with another author (SM) to check consistency, refine the codes, and ensure that the analysis was not influenced by our previous work (a systematic literature review [35]). The remaining data were coded, and codes and interpretations refined and reviewed as the analysis progressed. The codes ranged from descriptive to interpretative, capturing key concepts and ideas in the data, and were not clustered until the subsequent analysis phase. The data in the codes ranged from partial sentence fragments through complete sentences to entire paragraphs, with text segments allowed to fit into at least one code. After all data were coded, the text in all codes was cross-checked for consistency, further refining the generated codes.

Third, codes were grouped into initial clusters representing patterns of shared meaning using a whiteboard function in an online software tool Miro (miro.com). All authors discussed the draft themes in light of perspectives and experiences gained through the Policy development and implementation in monthly virtual Network meetings, and results and observations from the remaining parts of the HYPE study (organisational policy analysis [28], food and drink availability audits (Ni Mhurchu et al., submitted for publication), and staff and visitor surveys [39]). A draft summary of the initial themes was reviewed by SM and CNM and used in the fourth phase to further analyse codes and themes to generate explanations related to the research questions. Fifth, the themes were finalised, named, and their meaning, scope, and focus defined and agreed upon by all authors. Sixth, interview extracts were used as supportive quotes for the final themes and subthemes that are described in detail in the results section.

### Potential limitations of the research design and methods

Participant recruitment via email, which relies on obtaining a list of contacts, may result in a low response rate due to emails being overlooked or considered spam, and may introduce non-response bias, impacting the representativeness of the sample. Prior successful communication with the Network members via emails, and the overall preference for email communication indicated by food providers during food and drink audits as part of the HYPE study, justified the consistent use of this method.

## Results

### Participant characteristics

Thirty-three nominated participants were invited to take part in the interviews, of whom six declined to participate due to Covid-19, lack of time or staff shortages, seven indicated willingness to participate but did not respond after two follow-ups, and eight provided no response after three invitation attempts. The study sample comprised eight food providers and four Network members. Two food providers from the same company asked to participate in a joint interview. Three participants were from small (< 100,000 people), four from medium (100,000-300,000 people) and five from large (> 300,000 people) health districts. The interviews took on average 71 min (range 58–94 min) to complete. Three food providers and one Network member participated via email. The majority of participants had been in their roles between two and five years (range 1-14.5 years), although many were not in their current roles when the Policy was first adopted.

### Reflexive thematic analysis

Three themes were generated encompassing the experiences of the Policy implementers (food providers, FP, and Network members, NM). Tools and resources identified by the participants that could assist with the Policy implementation are described within the themes as appropriate.

### Theme 1: Complexities of operating food outlets under a healthy food and drink policy in public health sector settings

In general, food providers were described as having “*good intentions*” (NM#3) to implement the Policy, and they spoke about being “*100% behind in making things healthy*” (FP#2). However, the implementers described several complexities of operating food outlets in a hospital compared to operating cafes or restaurants in other settings.

#### Subtheme: Operating under several different food-related policies and contracts while catering to many distinct customer groups is challenging

Food providers operated simultaneously according to several food-related policies and contracts and often worked in different hospitals across the region within their DHBs. Food providers, especially in smaller DHBs, were often simultaneously responsible for running the inpatient meal service, providing Meals on Wheels in the community, providing meals in the staff cafeteria, operating public cafes and vending machines, and providing catering on hospital premises. The contrasts between the specific and strict nutritional, food safety and allergen regulations for inpatient and Meals on Wheels services, and the Policy nutritional criteria, were sometimes challenging to manage when preparing patient and staff café meals in the same kitchen. Food service personnel were able to adapt to the Policy requirements but needed time and training to make adjustments.

*“There’s lots of restrictions to [patient meals]. (…) we make it easy for ourselves, and we make kind of this ‘one pot wonder’ that then gets little things added to it [to be sold in cafeteria]. (…) It takes them [cooks] out of their comfort zone of what they’ve always been used to. But, they’ll get there, they’ve got there, that’s fine.”* (FP#6)

The implementers spoke about the differences between DHBs with respect to their population profiles [40], the variation in adopted healthy food and drink policy [28], the number of food outlets within the organisation, and whether the food outlets were operated internally or by external providers. From an organisational perspective, working in a large DHB with several external and commercial food providers, including franchised outlets expected to provide their usual core offerings, was challenging due to “*different businesses who have really different ways of working - or you know - different priorities around them*” (NM#3). In a smaller DHB, an operating model for the Policy implementation could have fewer food providers or only outlets operating internally. DHBs with a potential commercial interest in selling less healthy items, where the percentage of revenue was used to contribute to running other DHB services, was seen as conflicting for the Policy implementation.

An important contract with a bearing on the Policy implementation was the Multi-Employer Collective Agreement, which states that unionised resident medical officers are entitled to free meal(s) during their working hours at the expense of the DHBs [41]. As one participant noted, “*We talked quite a bit [about] becoming a sugar-free DHB. But some initial conversations with the doctors’ unions, made us realise that would be much more difficult than what we anticipated*.” (NM#2) The difficulty was mainly due to legal clauses that guarantee provision of certain items under the collective agreement that could not be restricted by the Policy.

Hospitals also catered to multiple distinct customer groups. Medical staff, including shift workers, often face challenges with long working hours in a highly demanding environment [13, 14]. High levels of stress are also experienced by visitors of unwell and sick patients (although the Policy is not aimed at them, they may eat at hospital food outlets). Implementers noted that “*a lot of the time especially when staff and visitors are busy, or stressed, they tell us they just want some comfort food*” (FP#7) and the “*requests for comfort foods including chocolate and sugar-sweetened beverages*” (NM#4) were barriers to the Policy implementation. Customer survey results align with our findings in that the second most common reason staff opposed the Policy (*n* = 221, 48%) was the removal of foods that provide energy or comfort [39]. Additionally, approximately one-fifth of visitors (*n* = 44, 23%) reported that the comfort feeling provided by foods/drinks influenced their product choice [39].

Participants saw that the Policy served as a guideline to follow and implement regardless of personal opinions. “*At the end of the day we are a contracted service – my client (the [DHB]) decided to implement the policy – we are just here to follow their directive*.” (FP#3) There was also appreciation that “*we got to look at this thing [the Policy] across the whole board, you know. We can’t just put a nutrition lens on it, we’ve got to be looking at it from the equity and accessibility lens as well and thinking what’s the right thing to do here*.” (NM#3) The introduction of healthier options was often associated with the perception that they were more expensive, less tasty, and unsuitable for sale in hospitals located in socio-economically disadvantaged areas.

#### Subtheme: Lack of a level playing field threatens food providers’ profits

Implementers noted there was an uneven playing field between food providers, particularly with respect to profitability. There were no consequences or incentives specified in contracts if food providers complied or did not comply with the Policy, even where contracts contained a Policy clause. For food providers implementing the Policy, business disadvantages were perceived as outweighing any possible benefits from being compliant, because customers could easily access and purchase ‘not permitted’ Red items (fried food, confectionery, and SSBs) nearby, e.g., just outside the hospital, and Network members had no authority to enforce compliance.

*“The reality is almost every hospital has a bakery or dairy within sight of the front door and to them the food policy is an absolute God send – because staff and visitors spend a fortune with them buying what we can’t sell (…) if I had the money I would buy a dairy as close to the hospital as I could get because I am afraid the healthy food policy just drives customers to their business.”* (FP#3)

The Policy was perceived as too restrictive to ensure the long-term profitability and sustainability of a commercial operation. The number of customers seeking healthier options was unlikely to ensure sustained profit margins for food providers adhering to the Policy. “*Well we obviously can’t sell what we want to sell - which reduces our takings. So everything that we do sell - the cost of goods is going to be spot on, because otherwise you’re losing money*.” (FP#4) A drop in profits was observed after the initial adoption and implementation of the Policy, and sales did not recover over time. Lesser profits from drinks were seen where the adopted policy only permitted plain water and milk, even more so when water was freely available from water fountains in the hospitals.

*“It definitely hurts the pocket financially. We have to work a lot harder than the average café to make our money. We can’t rely on the easy sale of a bottle of orange juice etc. We know from our non-DHB café sites that the drink sales, non-water are easy wins.” (FP#7)*

The decrease in profits in one food or drink category was predicted to have a ripple effect on sales of items in other categories, potentially decreasing the likelihood of retaining customers, impacting commercial viability, and threatening survival in a highly competitive market.

*“It is not just the juice that you [are] losing. Because that customer, potentially, would have (…) bought something else with that juice as a meal. So now, (…) you’ve taken not just the juice, you’ve taken the meal away as well.”* (FP#2)

Additional complexities were reflected in a mismatch between items permitted under the Policy and the current NZ food supply. Changing and adjusting recipes and mixed meals cooked on-site was perceived as feasible, although healthier ingredients were often not available in sufficient quantities and were more expensive, increasing the price of compliant menu items, sometimes already priced higher to make up for lost profits elsewhere. Preliminary audit results of food and drink availability in NZ hospitals confirm the higher price point for compliant Green items, which cost significantly more on average per item (NZ$6.00) than either Amber (NZ$4.70) or Red (NZ$4.00) foods/drinks (Ni Mhurchu et al., submitted for publication).

*“Commercial quantities of wholemeal pasta is a nightmare (…) there’s a limit to what I can do with 3 pasta shapes to keep the menu viable. So we do use non-wholemeal pasta as well. I suspect there just isn’t the market for a wide range of wholemeal pasta in commercial quantities and a dozen hospitals nationally asking for it isn’t likely to generate it either.”* (FP#3)

Food providers agreed that a tool to help assess recipe compliance was unlikely to be helpful or practical because it would be time-consuming to type in all ingredients and because recipes changed frequently. “*I don’t know if anyone’s going to have the time or the inclination to type in a recipe and see whether that recipe is compliant, or what parts are or aren’t*.” (FP#6) However, a tool to indicate if individual ingredients were policy-compliant or providing ready-to-use recipes could be useful.

It was perceived that some suppliers and manufacturers were unaware that the Policy existed, although many interviewees had a positive relationship with food suppliers and conveyed the requirements of the Policy to them. Small manufacturers with compliant products often struggled to keep up with the demand. Finding healthier and affordable packaged foods and snacks was problematic, even though some compliant products were available in the market (e.g., those produced to comply with school policy criteria). There was a need for a good understanding of the Policy criteria and time was required to search online or attend food shows to identify compliant products.

Implementers suggested creating a centralised database of packaged products with their traffic light classification to reduce the workload of individual food providers when searching for products, and to create an information-sharing platform for suppliers interested in offering their healthier items to hospital food providers.

*“Food providers and Policy monitors would benefit from having access to a living (continually updated) national database of commercial food/drink products with their Policy classification (Green/Amber/Red). This database would support consistent Policy implementation and monitoring across DHBs. It could also be used to promote a wider range of Green/Amber products to food providers (an incentive for food manufacturers to supply data, and for wholesalers to include Green/Amber products in food procurement systems).”* (NM#4)

Network members previously created a limited list of available packaged products, although this database became quickly outdated due to the continuously changing food supply.

### Theme 2: Adoption, implementation and monitoring of the Policy as a series of incoherent ad-hoc actions

This theme focused on various components of a healthy food policy cycle that were often incoherent and not well-coordinated, leading to inefficient and inconsistent actions. There were differences in experiences between the DHBs. Although participants expressed some uncertainties surrounding the new health reform and healthcare structure, several hoped it would bring more coordinated and nationally-led implementation.

#### Subtheme: The policy has not been sufficiently prioritised

In some DHBs, management was perceived as reluctant to sign off and officially approve the Policy or its adapted version. This hesitancy prevented implementation actions, as there was no official policy document in place. “*I don’t understand what the difficulty, what the delay in approving this is? (..) I think that’s a big support - just being able to, you got a piece of paper, and saying this is what we’re doing*.” (NM#1) Having management endorsement simplified matters when addressing customer enquiries about changes in food and drink availability. “*It’s nice for me to have that endorsement, because then it’s not just coming from me and it’s not my decision, and that was a lot of it*.” (FP#6) However, frequently no clear roles or responsibilities were assigned to individuals to oversee implementation or carry out monitoring, regardless of policy adopted by the DHB.

The Network was perceived by members as an important peer support group and its monthly meetings provided a platform to share individual successes, challenges, and feedback from food providers, and to learn from the experiences of other DHBs. Some DHBs allocated a proportion of the work hours of Network members or other public health staff to support the Policy implementation. In most cases, DHB employees who were ‘passionate’ about public health voluntarily took on the additional Policy-related work. Often, the amount of work was overwhelming for one person or a small unit to manage on top of other duties.

*“We don’t have a nutrition programme within our public health unit. And it has been no mandate or no real clear expectations from our DHB that [we] would be responsible for the Healthy Food and Drink Policy. (…) it would require some, you know, FTE [full-time equivalent] being dedicated to it.”* (NM#2)

One suggestion was to specify in employee workplans and contracts the responsibilities and number of hours that should be dedicated to implementation and monitoring the Policy regionally and nationally, and provide an online monitoring tool and associated training that would allow systematic, regular and feasible auditing of hospital food availability. Building public health nutrition capacity in each region was considered a priority so that policy-related work continued despite staff turnover and other work often prioritised in the DHBs, such as inpatient meal service, environmental sustainability initiatives, and time-critical issues (e.g., Covid–19 pandemic).

*“I’m one of the very few people on the Network, who has any capacity to work in this space. And I think it’s, you know, it’s one of the biggest downfalls of the National Policy, is that nobody’s actually got the capacity to work with it.”* (NM#3)

Clear and consistent regional capacity and streamlined implementation appeared more likely if a single policy was mandated and endorsed at the national level. Food providers would also welcome a nationally-led and consistent policy to create a more level playing field and demonstrate that the Policy is a priority for the government and DHB management. A consistent policy could also incentivise manufacturers to increase the number of policy-compliant products they offer because of increased demand in the market.

#### Subtheme: Engagement and collaboration between decision makers, implementers, and customers lacks transparency

Several interviewees alluded to a general lack of engagement and collaboration between implementers and decision makers during the adoption and implementation stages, which was sometimes linked to the Policy being voluntary and not officially endorsed by the Ministry of Health. Some perceived the adoption as a directive by the DHB management. “*The way it was communicated to our catering supervisor was pretty much: (…) this is the policy, and you have to follow it. Yeah, so quite a directive approach*.” (NM#2) At the same time, food providers’ concerns regarding profitability and implementation were dismissed. “*We wanted to ask questions about (…) how it was gonna be implemented, and the response was (…) “There is no discussion about this, you will take it or you will leave it, this is it”*.” (FP#2).

General lack of funding for implementation, and no financial incentives for complying with the Policy, meant Network members “*had to work really hard on the relationship aspect of implementing the Policy*.” (NM#3) Good working relationships were built with food providers in some DHBs but often undermined when Network members or workplace management demanded compliance and focused on minor non-compliance issues (which was perceived as a waste of time) rather than focusing on elements that had potential to impact on customers’ healthy eating habits more broadly.

Some DHBs had implementation plans in place, but often these were out of date, with some original implementation goals set years earlier still not achieved. A stepwise approach would likely support smooth implementation, as shown by one DHB that received help from a local public health unit in identifying healthier and compliant options.

*“So we went through the policy and had a look at it and thought, what can we do straight away, what’s going to take time, how are we going to communicate to our staff and our public? Because I knew it was going to meet some resistance, which it did. So we tried to work out the easy things first – and then put plans in place, where we could say ‘okay you’ve got three months to wean them off the 200 gram pies and put them onto, you know, smaller ones’.”* (FP#6)

There was also little consultation with hospital staff during the development and adoption of the Policy and, subsequently, insufficient, sporadic and reluctant communication about the changes during implementation from the DHB management. One participant noted, “*the DHB handle that side as it is their policy and their staff café – I don’t see that very much is done at all*” (FP#4). Some participants thought that “*it needs to be a national rollout and it needs to be talked, spoken about nationally. So if there is a national policy make it a national drive. So that people are aware of it*.” (FP#2) This led to discontent among the food providers, who felt the burden of communication, which they saw as the DHB’s responsibility, was pushed onto them and often involved dealing with customer complaints, verbal abuse and blame directed at food providers and their staff.

*“I can’t and I don’t expect all my staff to explain all this [the unavailability of some food and drinks] to customers. (…) the message should pass on to the visitors by putting a sign on the entrance promoting [the Policy] (…) so the customer will have that expectation that they won’t get a coke in the hospital.”* (FP#1)

*“I believe it has affected staff attitudes as we are the ones who receive the disgruntled feedback and complaints about no longer being able to purchase certain items.”* (FP#8)

Some DHBs had basic customer communication plans, often not (fully) enacted. One interviewee described a very successful, active and engaging communication effort driven by a local public health unit when the DHB changed to offering only healthy beverages. Although customer survey results indicated a high proportion of staff (*n* = 1986, 79%) and just over half of visitors (*n* = 142, 56%) were aware of the Policy, free text responses highlighted the need for improved communication with customers about the specific changes to hospital food environments to ensure their buy-in and engagement, and to promote healthier options [39]. In line with customer survey findings, interview participants also recommended improving communication with staff and visitors by using simple messages in the form of videos, posters, and flyers to communicate changes in food and drink availability, and explain the purpose and reasoning behind focusing on food environments rather than individual responsibility for healthier eating.

### Theme 3: Policy is (currently) not achieving the desired impact

In general, the implementers believed that DHBs should provide healthy options for staff and visitors and be role models for healthy eating for the wider community, and accepted the Policy, at least in principle, as a path to achieving this goal. “*I totally understand and applaud this policy. In its essence it is a wonderful concept and as a hospital we should be encouraging healthy eating habits*.” (FP#7). Customer survey results indicated that both staff (*n* = 1635, 66%) and visitors (*n* = 190, 76%) supported having a healthy food and drink policy in NZ hospitals. Among staff, the main reason for support was role modelling, as indicated by 82% (*n* = 1338) of respondents [39]. However, there was some doubt in our study whether the current Policy criteria accurately reflected the principles of healthy eating. This scepticism often stemmed from personal beliefs, variable knowledge and understanding of nutrition, and the many factors influencing individual food choices apart from healthiness.

There was a belief that if the Policy was implemented fully, it could have a positive and tangible impact on staff and visitors. “*I personally think it’s something really worthwhile, but it’s, hasn’t really flown and it has not been seen a priority within our organisation*.” (NM#2) Some interviewees acknowledged that they “*worked with quite a few different food and drink policies over the years, and none of them are perfect by any means*.” (NM#3) Overall, participants noted that the challenges outlined in themes 1 and 2 would need to be addressed for the Policy to have its desired positive impact, but also highlighted the importance of the factors mentioned in the following subtheme that play a role in healthy eating choices.

#### Subtheme: Unhealthy options remain highly accessible and attractive to hospital staff and visitors

Participants had conflicting views about consumer demand for healthier options. Some thought there was a demand for healthier options, which may have increased in recent years. “*I think so, yeah. People do like healthy food, I like healthy food myself*.” (FP#4) and “*I think people are always looking to eat healthily*.” (FP#5), but customers seeking healthier options were perceived as only a relatively small proportion of the current market.

*“From my experience, I think that there is a small portion [of people] that are really looking for some healthier alternatives. Most of the time, I get asked for less healthy food. Some people [are] asking, do you have this, do you have that, but most of the time, it’s less healthy food.”* (FP#1)

In some cases, customers who indicated they would like healthier options were considered unreliable when it came to purchasing food that aligned with their stated demand. Food providers were reluctant to continue to supply healthier options in the future if they did not sell well, especially when considering wastage and loss of profits from unsold healthier options.

*“When we put out the different healthy options we can get great feedback, but this is not reflected in the purchase. Too many say ‘oh this is nice’ or ‘what a great idea’ but do not buy. We were putting out a great range of salad boxes freshly prepared each day. We have had to stop because they didn’t sell; but were nice to look at! (…) We have a small core group of people who want the healthier options, but this is not always regular buying.” (FP#7)*

Some food providers also felt uncomfortable ‘pushing’ healthier options onto their customers and thought they should be free to sell a broader range of items than the Policy allowed and customers should be free to choose what they want to eat.

*“But when you’re talking, oh it can’t be over 120 grams this or it can’t be that, and I’m thinking, you know, who are you kidding? I mean, you’re telling grown people either what they can eat and what they can’t, you know. They’re not allowed a choice, and I think that’s wrong.”* (FP#4)

Generally, individual choice was a key argument against the Policy, often also voiced by customers. Customer survey results also showed that among staff who opposed the Policy (*n* = 465, 18%), the most common reason was the desire for the freedom to eat what they wanted (*n* = 313, 67%). However, only 15% (*n* = 71) agreed with the statement that the Policy will be ineffective in positively influencing food and drink choices in NZ hospitals [39]. Some food providers thought that instead of ‘policing’ what the customers could eat, “*there should be more education provided and better informed decisions made by customers rather than just removing their choices*.” (FP#8) Some food providers succeeded by making subtle changes to their menus and products but hesitated to promote these changes in case customers perceived healthier foods as less tasty. “*I don’t think people actually realise the changes that we have made, because we’ve made them kind of under the radar a little bit. And they haven’t had to miss out on anything*.” (FP#6) Another DHB successfully achieved a higher proportion of healthy items because they “*adopted a ‘quality improvement’ model (increase % of Green items, rather than strict Policy compliance), which food providers appreciated. However, a stricter approach was used to observe compliance with the subsequent soft drink ban*.” (NM#4) More collaboration and communication with food providers (and customers) could increase their understanding and buy-in of the Policy as a worthwhile food environment intervention.

*“It’s a challenge at times to simply explain to people why we want to influence the environment (…) It’s just sort of understanding that, you know, a lot of people seem to think it’s about individual choice, and so it’s moving people beyond that individual choice paradigm, I guess, and some want to hear that, and some don’t want to bother about that.”* (NM#3)

There was also a view that customers would not make healthier choices outside the DHB premises. Efforts of food providers to offer healthier options may have a limited impact on the health of staff and visitors because unhealthy options were readily available close to hospitals or through online food delivery platforms. “*I don’t think that’s going to make one iota’s difference in the bigger picture of things*.” (FP#2) Some commented that a significant change in the entire food supply would be required to see a positive difference in the customers’ health outcomes.

*“I’m too small. Doesn’t matter how hard I push. Doesn’t matter, what I do, I will not change one person’s eating habit. (…) If New Zealand really want to make the whole country compliant, (…) go to supermarkets, take out all the sugar in drinks. There will be much more useful than my little store.”* (FP#1)

Customers (sometimes even the health-conscious customers) who wanted confectionery, SSBs and deep-fried foods were perceived as willing to go and seek them elsewhere. Customer survey results indicated that although more than half of staff (52%) purchased food or drinks regularly (at least once a week) from food outlets within the healthcare facilities, a significant proportion of staff (42%) also bought items from food outlets outside the hospital at least once a week [39].

Some interview participants noticed an increase in purchases of unhealthy items that were subsequently brought onto the DHB premises, which could contribute to mixed messages to customers about the options available in hospitals. However, the changes in food and drink within the hospital might positively influence customers’ choices ‘by default’ because unhealthy options were no longer available.

*“So it’s about sending those messages. And it’s nice now when you’re down there, and a little kiddie wants a drink, and their options are milk and water. So you at least know, they’re going to have that healthy options.”* (FP#6)

The key to customer buy-in and increasing their demand for healthier options offered by DHB food providers was believed to be ensuring that tasty, familiar, and well-presented food was available.

## Discussion

This study outlines the experiences of food providers and Food and Drink Environments Network members in adopting and implementing the National Healthy Food and Drink Policy in NZ. Several challenges and barriers were identified, broadly summarised as little coherent action to advance the Policy implementation nationally, insufficient hands-on local support for individual food providers’ needs, inadequate consideration of the hospital operating landscape and its diverse customer groups, substantive effort and knowledge required to identify compliant products, and the ability to purchase unhealthy foods in the nearby vicinity of hospital premises. Similar challenges were previously identified for public sector workplaces [35], schools [42], recreation centres [43], and other retail outlets interventions in public sector settings [44]. Thus, the NZ Policy is not unique in failing to be successfully implemented, and it is unlikely that successful implementation would happen without careful planning, adequate support and funding, and an increase in public demand for healthier options.

Profitability, closely related to customer demand for healthier options, and the proximity of readily available and desirable unhealthy options prohibited under the adopted policy, was unsurprisingly a major concern for food providers, reflecting findings in the international literature [35, 42, 45–47]. Several factors may contribute to profit loss when a healthy food and drink policy is introduced and implemented. Currently, there is no clear guidance or evidence on how to mitigate these negative financial impacts in public sector settings or how to financially incentivise food providers to implement and comply with the adopted policy. While price incentives for healthier options have been shown to significantly increase purchases among hospital employees [48], research on financial incentives at the food provider level is lacking. The World Health Organization’s action framework for implementing healthy food policies in public settings [16] suggests incentives such as increased publicity for compliance, meeting supply demand for a policy adopted across multiple institutions, and eligibility for food procurement contracts. In North Carolina, non-monetary reward (‘Red Apple’ status) incentivised hospital food providers to fully implement a healthy food environment project [49]. Adopting similar schemes could positively incentivise the implementation of the NZ Policy.

Our study did not explore the views of food manufacturers and suppliers. However, interview participants mentioned having good relationships with these stakeholders, which facilitated implementation. The exact nature and extent of interactions between food providers and Network members with suppliers and manufacturers were not examined. Some manufacturers in NZ responded to market demand by reformulating products, producing healthier options, or offering smaller packages. This aligns with findings from a study in British Columbia, Canada, where food manufacturers were incentivised to produce policy-compliant items due to an increase in the number of publicly funded recreation facilities adopting uniform guidelines [50]. For larger manufacturers in NZ, the Policy did not create enough market share to invest in producing compliant products to offset the high costs associated with healthier reformulation and production of policy-compliant items [51, 52].

Manufacturers may be more inclined to undertake product changes when there is strong support and commitment from governments and organisation leadership to adopt a mandatory, endorsed, and consistent food policy across all institutions [51, 52]. Such a policy provides the legal basis for enforcing compliance (especially when contract clauses are used [16]), signals the policy’s importance to all stakeholders, creates a level playing field among food providers, and justifies allocation of resources and funding for its adoption and implementation [35]. Mandatory policies were effective at improving healthiness in public institutions in New South Wales, Australia [18], Scotland [19], and Washington State, USA [53], in contrast to the voluntary NZ Policy, which showed that 39% of products in hospitals was classified as Red (not permitted) and only 22% as Green (target ≥ 55%) during the 2021/22 food/drink availability audits (Ni Mhurchu et al., submitted for publication). However, mandatory policies may not be feasible in all public settings or difficult to legislate at a national level [3]. Nevertheless, voluntary but well-supported policies can also lead to a successful implementation [16]. Yet, the voluntary Policy in NZ was not mandated [24], was not consistently adopted [28], and has not been supported by tailored tools or resources [26].

Absence of adequate tools and resources to support implementation of the Policy was previously identified [26]. Participants in this study suggested two tools could significantly support the Policy going forward. The first was a digital auditing tool to monitor compliance. Web-based tools for policy monitoring have been used in New South Wales [18] and Victoria [54] in Australia, and developed for the HYPE study in NZ to collate on-site food and drink data [55]. A second supportive tool recommended by the participants was an online database of compliant packaged foods and drinks, allowing food providers to quickly and easily find suitable products available on the NZ market. Similar tools are available in New South Wales [56], Victoria [54], and Canada [57]. This tool could also facilitate communication between DHB food providers and external food suppliers and manufacturers about suitable available products. Ongoing government funding of these supportive tools would be required to maintain the online platforms [58] and regularly update the product database [59].

Although the implementation of healthy food policies is generally considered challenging in a free market economy [29], the development and adoption of government-led guidelines is a critical and fundamental process in the food policy cycle [60]. This process was outlined for Washington State, US food service guidelines, where one of the recommendations was “*including both the public health and business perspective (i.e., businesses still need to make a profit)*” [61 (p.54)]. The financial aspects in this study were closely related to customer buy-in and their demand for healthier options, and the proximity of readily available and desirable unhealthy options prohibited under the adopted policy.

A prevalent view in this study was the need for freedom of choice to sell and purchase unrestricted items in commercial settings, which the Policy was perceived as restricting. Individual responsibility for one’s own food choices and health is common in neoliberal societies such as NZ [62]. For customers who wanted to make healthier choices, and those nudged towards less healthy choices via marketing techniques or an unsupportive food environment [63–65], the Policy was considered to support customers to make healthier choices by counteracting ubiquitous unhealthy food and drinks [66, 67]. Shifting cultural norms surrounding eating practices at a population level is highly challenging and complex [68], especially in stressful work environments [64, 69], and food providers in this study felt this responsibility rested solely with them, with minimal government efforts to drive a national cultural shift towards healthier options.

### Policy and practice recommendations

The following are key recommendations to support food providers and Network members in successfully implementing the Policy, which can also be applied more broadly to other healthy food and drink policies in various settings.

- Adoption of a single national policy mandated by government, combined with legally binding contract clauses, to create a level playing field between hospital food providers and send consistent messages on healthy eating to staff and visitors.
- Offering of financial incentives for food providers who achieve compliance with the Policy (e.g., more favourable lease conditions).
- Better engagement with on-site food providers, including co-developing and testing of initiatives within a policy [70] to decrease the potential negative business outcomes associated with policy implementation.
- More regular, ongoing, and frequent communication with staff and visitors from DHBs and other government agencies, (e.g., positive messaging around healthy eating and non-health related benefits such as environmental sustainability) and communicating via various channels to increase buy-in and catalyse change.
- Use of strategies to make healthier choices more attractive and increase customer demand, e.g., tasting sessions for new healthy products, customer surveys to identify desirable healthier options, competitively pricing healthier items, and loyalty programmes for healthier purchases [35].
- Provision of adequate funding and support to implementers, including the development and maintenance of tools, to assist with adoption, implementation and regular monitoring of the Policy.

### Future research directions

This study suggests future research directions for the implementation and compliance with healthy food and drink policies in public sector settings. First, investigating attributes of policy frameworks that are more feasible to implement could reduce the associated challenges and the need for supportive tools and resources. Second, longitudinal research, similar to the annual evaluations of the mandatory Washington State’s Healthy Nutrition Guidelines from 2014 to 2018 [53], is recommended to monitor changes over time. Third, further research should focus on strategies to increase and maintain customer demand for policy-compliant healthy food and drink options and mitigate profit losses associated with introducing healthy food and drink policies. Last, gaining insights from the food supply industry and government representatives could provide additional perspectives from key stakeholder groups.

### Strengths and limitations

This is the first study reporting on the experiences of hospital food providers and public health dietitians/staff in implementing the voluntary NZ Policy. The interview questions were based on a previously used interview guide and modified for the NZ context using relevant research findings. Data collection and analysis were rigorous, and findings were interpreted in light of other HYPE study results (specified in the methods section) and insights from the Network, allowing for an in-depth Policy case study. However, there were some limitations. First, the interview sample included food providers and Network members only. It is likely that other stakeholders, such as government representatives and food suppliers, would have added different perspectives and insights, and including them in future studies and evaluations is recommended. Second, four out of twelve participants completed their interviews by email, and an inconsistent approach to interviewing could introduce some bias to the study, although our findings are similar to those previously reported in the literature [35]. Third, the small sample of 12 participants was due to a limited pool of potential participants and the Covid-19 pandemic that significantly affected all staff in healthcare facilities at that time. Future studies could use another form of data collection from food providers, such as quantitative surveys, to reduce researcher and individual participant burden, although these methods may not capture in-depth context and insights [31].

## Conclusions

The voluntary nature and inconsistent adoption of the Policy, the presence of food outlets close to hospitals serving unhealthy foods, and a culture of unhealthy eating, combined with the difficulty in changing people’s eating habits, are challenges to the implementation of the Policy and important barriers to overcome. Key recommendations to promote successful Policy implementation include the adoption of a mandatory national Policy, offering incentives for achieving compliance, better engagement with food providers, good communication with staff and visitors using positive messaging, provision of funding for implementation, and availability of a food monitoring tool and a searchable database of policy-compliant products. Findings of this study could inform updates to the Policy and development of suitable supportive tools, and improve the adoption and implementation of similar policies for other settings.

## Electronic supplementary material

Below is the link to the electronic supplementary material.

Supplementary Material 1

## Data Availability

The interview data analysed in this study is not publicly available due to presence of sensitive and identifiable information in the qualitative data that could compromise the anonymity of the participants, potentially subjecting them to psychological or social risks. Additionally, participants in this study did not give consent for their data to be shared publicly. However, the corresponding author may grant access to specific anonymised datasets upon reasonable request.

## Abbreviations

DHB: District Health Board
FP: Food Provider
HYPE: HealthY Policy Evaluation
NZ: New Zealand
SSB: Sugar Sweetened Beverage
NM: Network Member
