# Supplementary material for: Implementing healthy food policies in health sector settings: New Zealand stakeholder perspectives
Source: BMC Nutr. 2024 Sep 7;10:119. doi: 10.1186/s40795-024-00924-z (PMC11380432; doi:10.1186/s40795-024-00924-z)
Supplement: Supplementary file 1 — Supplementary Material 1 [file 40795_2024_924_MOESM1_ESM.docx]

**Implementing healthy food policies in health sector settings:
New Zealand stakeholder perspectives**

HYPE (HealthY Food Evaluation) Study Interview Guide

(FP) specific food provider prompts,

(NM) specific District Health Board (DHB) Network member prompts

Background Question(s)

1. Can you tell me about your role at the [name of DHB/organisation]?

- main responsibilities
- length in this role

Awareness and attitudes towards Policy and implementation

How has the adoption and implementation of the National Healthy Food and Drink Policy *(referred to as the Policy from here on)* been communicated (with you)?

- official endorsement of the Policy by the [name of DHB/organisation]
- implementation plan, milestones or goals
- dedicated point-of-contact person
- frequency and means/form of communication
- contract clause specifying that food providers must comply with the adopted Policy

Can you tell me about your experiences of implementing the Policy?

- feelings about implementation when first heard about it (FP)
- how implementation of the Policy fits into your role
- confidence in classifying foods or drinks into *Green*, *Amber* or *Red* categories
- initial response from food providers (NM)

Implementation processes

What practical steps or actions have you (or the DHB/organisation) taken so far towards implementation of the Policy?

- work done in relation to the Policy implementation
- Policy criteria easiest to implement
- Policy criteria most challenging to implement
- product placement/prominence changes to comply with display restrictions (FP)
- sourcing foods/drinks compliant with the Policy (FP)
- creating new recipes (FP)
- modifying existing recipes (FP)

Has the implementation of the Policy taken much of your time?

- increased workload
- additional responsibilities or tasks
- additional staff training
- additional contact with/support of suppliers

Facilitators and barriers

What do you think were the factors (if any) that facilitated or promoted the Policy implementation across the DHB/organisation facilities or by your company?

- successes of Policy implementation in own food outlet
- support available in [name of DHB/organisation]
- availability of nutritionists/dietitians or another dedicated person/role
- collaboration with other retailers or organisations
- training, workshops, resources, tools related to the Policy
- financial support or incentives

In your experience, what are the barriers or challenges (if any) to the Policy implementation across the DHB/organisation facilities or in your company?

- specific strategies used to overcome these barriers
- time required to make changes
- availability, range and cost of healthier products from suppliers
- shape/size/packaging type of healthier products (especially for vending machines)
- time to prepare healthier foods, lack of standardised recipes
- availability, range and cost of healthier products/ingredients from suppliers
- suppliers’ cooperation to implement the Policy
- equipment or space to prepare/store healthier foods and drinks
- feedback received from food providers on points above (NM)

Staff and visitors

What kind of feedback have you received from the staff or visitors about the Policy?

- staff or visitors asking about changes
- satisfaction with healthier options
- satisfaction with the removal of less healthy/familiar/unhealthy options
- requests for culturally appropriate foods and drinks
  (e.g., from Māori or Pasifika staff and visitors)
- requests for special dietary or religious requirement foods and drinks
  (e.g., vegetarian, vegan, dairy free, kosher, halal)

How do you think the adoption and implementation of the Policy has affected customers’ choices and food purchases?

- demand for healthier food and drink options
- purchasing of foods and drinks from outside the DHB/organisation

How have you, or the [name of DHB/organisation], promoted or communicated the Policy and healthier options to the staff and visitors?

- educational materials, posters, newsletters, email, social media
- encouragement from food outlet employees (FP)
- discounts or competitive pricing of *Green* and *Amber* options (FP)

Unintended or unforeseen consequences

What has been the impact of the Policy on your business (if any) following changes in food/drink offerings? (Or feedback received from food providers (NM))

- sales, profits
- standard business operations
- food outlet staff attitudes and turnover

Have there been any unintended or unforeseen consequences from the adoption and implementation of the Policy?

- increase or decrease in food waste or food packaging
- increase in staff and visitors bringing foods/drinks from outside or nearby retailers
- effect on working/business relationships

Further work and resources

What further work do you think is required in the [name of DHB/organisation] or your company to fully implement the Policy?

- particular Policy criteria proving hard to implement
- reporting on progress and implementation

In your opinion, what other kinds of resources or tools could help food retailers, such as yourself, with the Policy implementation?

- format (online/digital or paper-based)
- implementation guide, training videos, workshops
- tool to classify foods and drinks into *Green*, *Amber* and *Red* categories
- database listing suitable *Green* and *Amber* products
- menu planning tool, recipe analysis tool, healthier ingredients swap list, sample recipes
